# ELIXIR‐EXCELERATE: establishing Europe's data infrastructure for the life science research of the future

**DOI:** 10.15252/embj.2020107409

**Published:** 2021-02-09

**Authors:** Jennifer Harrow, John Hancock, ELIXIR‐EXCELERATE Community, Niklas Blomberg, Søren Brunak, Salvador Capella‐Gutierrez, Christine Durinx, Chris T. Evelo, Carole Goble, Ivo Gut, Jon Ison, Thomas Keane, Brane Leskošek, Luděk Matyska, Johanna McEntyre, Célia Miguel, Arcadi Navarro, Steven Newhouse, Tommi Nyrönen, Patricia Palagi, Bengt Persson, Cyril Pommier, Jordi Rambla, Marco Roos, Gabriella Rustici, Andrew Smith, Alfonso Valencia, Celia van Gelder, Jiri Vondrasek, Nils Peder Willassen, Juan Arenas, Helen Parkinson, Robert D Finn, Sergi Beltran, Leslie Matalonga, Hannah Hurst, Paul Kersey, Ilkka Lappalainen, Pascal Kahlem, Gary Saunders, Sirarat Sarntivijai, Rachel Drysdale, Johnathan Tedds, Jeremy Lanfear, Jennifer Harrow, Niklas Blomberg

**Affiliations:** ^1^ ELIXIR Hub, Wellcome Genome Campus Hinxton, Cambridge UK

**Keywords:** Computational Biology, Methods & Resources, S&S: Ethics

## Abstract

A new inter‐governmental research infrastructure, ELIXIR, aims to unify bioinformatics resources and life science data across Europe, thereby facilitating their mining and (re‐)use.

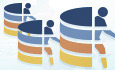

Creating knowledge by connecting and analysing large amounts of life science data is transforming our society, allowing us to start addressing major scientific and societal challenges, such as adaptation to climate change or pathogen outbreaks in an interconnected world. Modern biology is dependent on the generation, sharing and integrated analysis of digital data at scale. A deeper understanding of biological systems is now becoming possible thanks to breakthroughs in technologies that study life systematically at different scales, from molecules and single‐cell pathogens to complex animal or plant models and ecosystems as well as across temporal ranges spanning split‐second reactions to multi‐year clinical or agronomic trials, and beyond. The key to analyse and leverage this complex, fragmented and geographically dispersed life science data landscape is to ensure it is easy to find and reuse by researchers. This article comments on ELIXIR, an international organisation that brings together bioinformatics researchers and life science resources across Europe and integrates them into a single federated infrastructure.

## Addressing the data challenges of modern biology

At present, analysis often involves integrating large datasets from multiple sources. Life science archives are rapidly increasing in size and complexity; for example, the archives held by EMBL‐EBI double in size approximately every 2 years (Cook *et al*, 2016) so that long‐term data stewardship is vital. The chances of retrieving data from any given scientific publication may decline by as much as 17% per year (Vines *et al*, 2014). Data have been generated for different research purposes at thousands of facilities across the world and are captured and stored in diverse formats. This creates a significant barrier to data integration and reuse (Rigden *et al*, 2016), as well as necessitating a massive data storage and exchange burden (Cook *et al*, 2016). In addition, data need to remain accessible and be updated long term for future reuse. Over 1,000 data resources exist in Europe and over 5,000 worldwide [https://bigd.big.ac.cn/databasecommons/], but only a small fraction of these have institutional support and long‐term funding commitments (Imker, 2020). The fact that the mid‐ and long‐term sustainability of many crucial bioinformatics resources, such as UniProt [https://www.uniprot.org/], Ensembl [https://www.ensembl.org/], EGA [https://ega‐archive.org/] and Silva [https://www.arb‐silva.de/], is not guaranteed threatens the foundations of academic and industrial life science activities, risking the loss of an immense wealth of biological and biomedical information, and wasting those associated historical investments. To address these challenges, ELIXIR became operational in 2014. Intergovernmental by nature, it is funded by financial contributions from its member countries (each of which, along with EMBL‐EBI, hosts an ELIXIR Node), alongside other grants. Here, we describe the progress made by ELIXIR as a result of European Union’s €19 million ELIXIR‐EXCELERATE grant from 2015 to 2019. This funding was provided, following the ESFRI and European Council decision in 2014 to categorise ELIXIR as one of Europe’s three priority research infrastructures. A broader description of the ELIXIR Infrastructure, platforms and communities can be found in J. Harrow, R. Drysdale, A. Smith, S. Repo, J. Lanfear, and N. Blomberg (submitted). Here, we focus on the developments that have direct impact on users of bioinformatics services built on the ELIXIR infrastructure, funded through ELIXIR‐EXCELERATE.

**Figure 1:**
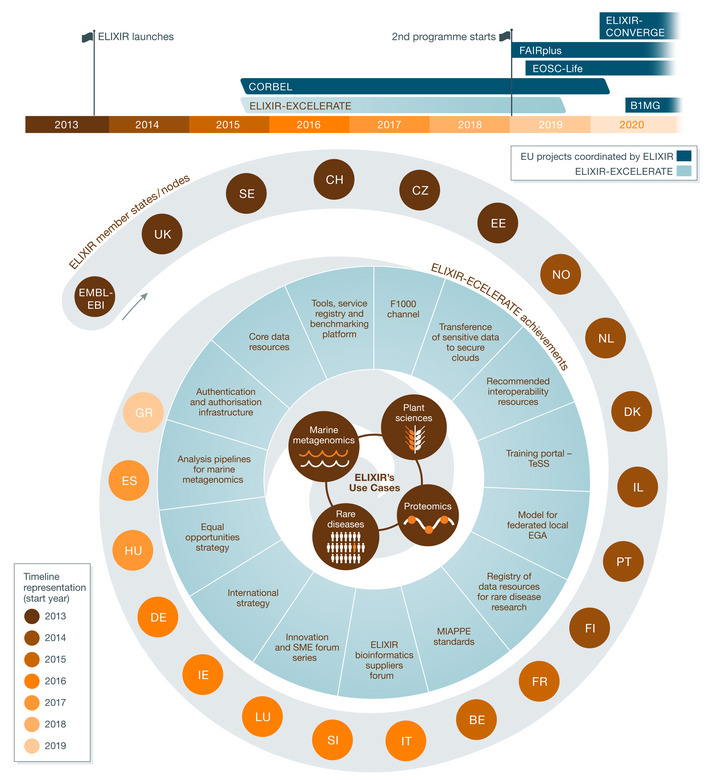
Development of ELIXIR as Europe's life science data infrastructure. Schematic overview of the establishment of ELIXIR, including the ELIXIR‐EXCELERATE achievements, a timeline of when members joined and an overview of Use Cases established during the launch of the ELIXIR‐EXCELERATE grant.

ELIXIR has worked to meet its key challenges around data sharing, reuse and resource sustainability by consolidating Europe’s national centres and bioinformatics resources into a coordinated infrastructure (both a technical network and a people network), operating as a distributed virtual organisation. Figure 1 highlights key milestones in its progress, such as the development of ELIXIR Communities, and partnership with the Global Alliance for Genomics and Health, as ELIXIR becomes established as a key European life science infrastructure and moves to a mature operational phase during its 2019–2023 scientific programme. ELIXIR’s successful development is underlined by the fact that it now brings together more than 220 institutes within 23 members (22 countries plus EMBL‐EBI), meeting the needs of over a half‐million life scientists across Europe. ELIXIR increasingly ensures that users (individual scientists, companies, large consortia and other research infrastructures) can easily access data resources, built on strong community standards and safeguarded over the long term. It has produced a concerted effort to connect national infrastructures that reach out to local and regional centres with Europe‐wide reference data resources and support services for data standards. By coordinating Europe’s national and international capabilities into a coherent infrastructure, our 500,000+ users will seamlessly navigate an ecosystem of life science data services.

In this publication, we summarise the development of ELIXIR (as depicted in Fig 1) in the context of the H2020 ELIXIR‐EXCELERATE project. We focus on describing how ELIXIR has developed mechanisms and foundations to align its operations with the FAIR principles, and how this supports the effective use of open life science data. We further illustrate how the expertise from individual ELIXIR member institutions has worked together to align national and international services into a standards‐based infrastructure, operating at a pan European scale.

## Building a stable and sustainable infrastructure for biological information across europe with the aid of ELIXIR‐EXCELERATE

Underlying the success of ELIXIR has been its ability to work with users in different domains of life science research. Researchers work on generic solutions that can also be applied in other communities; lessons learnt are then taken from a specific field and widen the uptake of those solutions to other unrelated fields, by sharing them across ELIXIR’s members and user communities. ELIXIR provides its expertise via five technical domains of implementation called Platforms—Compute, Data, Interoperability, Tools and Training. The headline outputs of the ELIXIR Platforms during EXCELERATE are summarised in Table 1. For more details, see J. Harrow, R. Drysdale, A. Smith, S. Repo, J. Lanfear, and N. Blomberg (submitted) and the individual Platform web pages [https://elixir‐europe.org/platforms].

**Table 1:** ELIXIR Platforms and major outputs from the ELIXIR‐EXCELERATE grant

Platform	Headline outputs
Compute	Developed the ELIXIR Authentication and Authorisation Infrastructure (ELIXIR AAI) Demonstrated technology to transfer sensitive human data to secure clouds Container orchestration Hybrid cloud capacities Integrated solutions for ELIXIR Communities
Data	Defined criteria for and identified the ELIXIR Core Data Resources Contributed to the establishment of Global BioData Coalition Infrastructure to support community annotation and linking
Interoperability	Identified the set of ELIXIR Recommended Interoperability resources Bioschemas: schema.org submission and adoption for life sciences CWL: workflow interoperability and adoption Developed framework deploying interoperability services Bring your own data and capacity‐building workshops
Tools	Developed ELIXIR Tools and Service registry (bio.tools) Developed EDAM ontology for the annotation of tools and services by the community Developed the ELIXIR benchmarking platform (OpenEBench) making DREAM challenges results available
Training	Delivered over 850 training events, to over 19,000 people across 60 countries Established TeSS, ELIXIR's Training Portal, a registry of training events and materials Consolidation and expansion of the network of training providers in Europe Established the ELIXIR Train‐the‐Trainer programme, E‐learning platform and Virtual Coffee Room Developed the ELIXIR Training Toolkit

Orthogonal to the Platforms is what have become known as the ELIXIR Communities (originally called Use Cases in ELIXIR‐EXCELERATE), which bring together individuals from across the ELIXIR members to identify and address specific issues relevant to that area. The first four Use Cases in ELIXIR‐EXCELERATE were Human Data, Rare Diseases, Marine Metagenomics and Plant Sciences, each with their own unique technical and legal challenges in addition for Human Data and Rare Diseases.

ELIXIR facilitates the optimal reuse of existing and future life science data by applying the FAIR principles (Wilkinson *et al*, 2016). Data must be findable, accessible, interoperable and, ultimately, reusable. The FAIR principles describe how data, including life science data, can be fully utilised by both humans and computer systems (see Box 1). Well‐managed research data in the life sciences generate value in the research community, industry, education and society at large, far beyond the initial researcher’s laboratory. For example, an impact report of the European Bioinformatics Institute showed that its value to researchers and funders was over 20 times its operational cost [https://beagrie.com/static/resource/EBI‐impact‐report.pdf]. In ELIXIR’s vision, the FAIR principles apply not only to the data, but also to the tools and workflows used to analyse and interoperate them, the training resources needed to build capacity internationally to analyse and manage the data, and the compute infrastructure needed to access and analyse data at scale.

Box 1 **ELIXIR has published and bases its work on the following guiding principles for FAIR Data Management in the life sciences (ref https://f1000research.com/documents/6‐1857):**

- Open sharing of research data is a core principle for publicly funded research and ELIXIR encourages all funders to adopt Open Data mandates and aims to support those mandates.
- Data Management is a crucial part of good scientific practice and research excellence and is being followed up in the CONVERGE project.
- Whenever possible, biological research data should be submitted to the recommended community deposition databases.
- All data submitted to Open Data archives must be annotated in accordance with community‐defined standards.
- ELIXIR members facilitate the national implementation of a harmonised FAIR Data Management programme for the life sciences.
- FAIR Data Management requires professional skills, reusable tools, services and workflows, and adequate resources.
- Good research data management requires appropriate funding for data infrastructures.

ELIXIR is an open infrastructure and does not “own” or operate data resources or other services. Rather, it provides a coordinated data and service backbone that allows partners (e.g. other Research Infrastructures [https://www.esfri.eu/health‐food], national resources, institutional archives) to make use of existing resources and connect and interoperate their own resources, building on service levels guaranteed by the ELIXIR branding. Ensuring interoperability between resources and data enables long‐term, cost‐effective data management and drives “standards as the default” across the life sciences. However, this also relies on the stability of key datasets that underlie data reuse in the life sciences.

In addition, ELIXIR‐EXCELERATE enabled a series of capacity‐building activities in emerging scientific areas, such as genome assembly and annotation, where six successful high‐level workshops were given across Europe, and single‐cell transcriptomics, later growing to form an ELIXIR user Community.

## Establishing an open data framework for European life science through ELIXIR infrastructure

Essential to FAIR data and optimal data reuse is a strategy for data management. A data management strategy defines how to handle the entire data lifecycle. ELIXIR underpins and drives good data management practice in the life sciences, and in future is committed to making data available within the framework of the European Open Science Cloud (EOSC) [https://www.eosc‐portal.eu/]. ELIXIR promotes open and free data access to the maximum extent possible, since it is difficult, if not impossible, to interoperate and integrate data across a complex web of licences and contractual limitations—discoveries get lost in legal red tape. ELIXIR recognises, however, that restrictions are needed for some data types, such as personal data. Charging for, or restricting access to data, seriously limits the ability of research organisations, both public and private, to exploit and create additional value from collective public research investments.

In the following sections, we describe concrete implementations and standards developed by ELIXIR during the ELIXIR‐EXCELERATE project, summarising the general principles that emerge from these examples and how they help both computer and bench scientists go about their work. This serves to underline the value of a large collaborative infrastructure in developing new services that can have direct benefit to any life scientist.

## Distributed search and access to plant phenotype datasets

The exploitation of modern genomics and phenotyping technologies is increasingly driving the development of new crops and commercial plant cultivars that are needed to address major challenges to be faced by agriculture such as adaptation to climate change, decreasing its environmental impacts and feeding the expanding population. Plant phenotype data are central to the development of new and improved crops and to identifying the genomic regions underlying particular traits. This type of data is difficult to find because there are no central repository and no plan to build one. Indeed, the heterogeneous nature of phenotype data led to the implementation of diverse infrastructures and experimental platforms backed by specialised data collection and management schemes, including dedicated ontologies used to describe phenotypes of interest within specific plant communities. Nevertheless, it is important that these datasets become FAIR to allow improved reproducibility and reusability across the different communities working with plant phenotype data. To address this, the ELIXIR Plant Sciences Community played a major role in extending and further developing the MIAPPE standard [https://www.miappe.org/] to describe plant phenotyping experiments (making the phenotype data more readily interpretable) and the Breeding API (BrAPI) standard [https://brapi.org/], which allows machine‐actionable access to disparate datasets. Based on these innovations, the Community has developed the FAIDARE BrAPI‐based portal [https://urgi.versailles.inra.fr/faidare/], a data discovery service to search relevant, ontology‐annotated datasets with linkage to the European Nucleotide Archive (ENA) [https://www.ebi.ac.uk/ena/browser/home]. The FAIDARE federation currently indexes eight resources, providing access to over 150,000 datasets (as of 3 September 2020). This work is led by the ELIXIR France Node, in collaboration with EMPHASIS, the plant phenotyping ESFRI, international groups such as the MIAPPE and BrAPI consortia, and with support from ELIXIR’s Interoperability Platform.

## Anonymised prioritisation of disease‐related genomic variants

Sharing sensitive human genomic data across borders is essential to gain an understanding of the genetic basis of diseases, especially in the case of rare diseases where large datasets are needed and single countries (particularly smaller countries with inherently smaller local datasets) do not have sufficient infrastructure to archive and distribute these data. Human genetic data raise specific issues with regard to findability, as data must not be identifiable (i.e. traceable to a single individual). To address this, the ELIXIR Federated Human Data Community is working with the Global Alliance for Genomics and Health (GA4GH) use the Beacon discovery service for resources across ELIXIR. A Beacon is defined as a web‐accessible service that can be queried for information about a specific allele, with no reference to a specific sample or patient (Fiume *et al*, 2019). Lightweight metadata provided by a data resource (a “Beacon”) can be interrogated to ask “Have you observed this nucleotide at this specific chromosomal position?”, and the query response is a “Yes” or “No” answer. A Beacon may serve data from case‐level observations, such as genetic variants identified from sequenced samples, or from annotation resources, such as variant–disease associations curated from scientific literature. Beacons represent an important step towards collaborative, responsible sharing of human genomic data, compatible with sharing information about identifiable data and the European GDPR regulation.

Work within ELIXIR has driven the establishment of a network of ELIXIR Beacons via strategic partnering with national data owners to enable data flow to the Beacon service. Development of the Beacon infrastructure has involved strong interactions with the ELIXIR Training, Compute and Interoperability Platforms, reflecting recognition that Beacons represent a simple and useful mechanism for data discovery. Our work further aims to increase the integration of the Beacon API with human data resources throughout ELIXIR and extend its application to other data resources, and currently, there are 42 international organisations using this API to serve > 1,000,000 anonymised human samples across 200 datasets.

## Privacy‐compliant access to human genomic data

The European Genome‐Phenome Archive (EGA) is a database infrastructure for archiving and distributing sensitive human genomic and phenomic data that, by definition, require controlled access. As with Beacons, the key issue for the EGA is to protect sample confidentiality while enabling research. The EGA complements Beacons by making confidential data accessible. EGA was founded in 2008, and in 2013, a collaboration was established to mirror the infrastructure at the Centre for Genomic Regulation (CRG) in Barcelona, part of ELIXIR—Spain. During ELIXIR‐EXCELERATE, ELIXIR supported improvements in the submission process to the EGA and the development of local EGA, an infrastructure allowing easy, local installation of EGA to be used for the collection of genome and phenome information at the national, regional or even institutional level.

**Figure 2:**
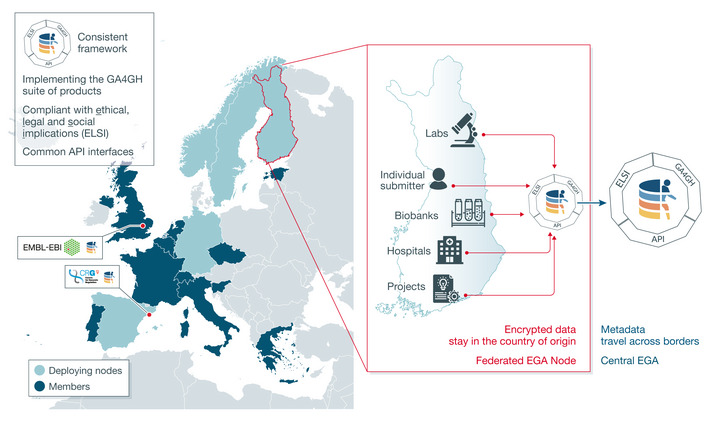
Federated access to European Genomes On the left, 17 of the 23 Nodes are members of the ELIXIR Federated Human Data Community. EMBL‐EBI (Cambridge, UK) and CRG (Barcelona, Spain) are specifically highlighted as these are the host institutes of the central EGA. Five Federated EGA deploying Nodes (Finland, Germany, Norway, Spain and Sweden) are also highlighted. These implemented the Federated EGA framework in the first wave to manage archival, access and analysis of sensitive human data. On the right, a schematic view of the ELIXIR Finland Federated EGA deployment. Sensitive human data generated at laboratories, BioBanks and hospitals, and/or by individual projects and submitters, are stored in encrypted format within the countries’ jurisdiction. These sensitive data never leave the Finnish borders. Metadata to describe the datasets is shared with the central EGA, which enables findability of these data. Authorised users are able to access these sensitive data remotely thanks to the suite of interoperable GA4GH standards.

Local EGA [https://ega‐archive.org/federated] allows deposition of sensitive human data locally (complying with national guidelines for storing that data), but enables data reuse across national boundaries. Local EGAs store metadata from the central EGA, allowing the use of the local EGA to search both the main and local EGA. Search and retrieval of information from the local EGA is also possible using the local EGA API, allowing the building of local services based on the data available. Additionally, the central EGA gathers non‐sensitive metadata (e.g. dataset descriptions) from all the data submitted to local EGAs, so a search at the central EGA allows data located across all local EGAs to be found. The local EGA activity links to the Compute, Training and Interoperability Platforms of ELIXIR. The collaborative work of local EGA instances with the central EGA is collectively known as the Federated EGA. Indeed, the Federated EGA (see Fig 2) is part of the European COVID‐19 Data Platform [https://www.covid19dataportal.org/], which has been established to facilitate the sharing of national SARS‐CoV‐2 viral and associated host sequence datasets.

## ELIXIR infrastructure for rare disease research

The ELIXIR‐EXCELERATE Rare Disease Community laid the foundation towards building a robust bioinformatics infrastructure at the European level for and with the rare diseases (RD) community. The catalogue of ELIXIR Rare Disease resources (https://rare‐diseases.bio.tools) was developed in collaboration with the ELIXIR Tools Platform and currently contains 133 relevant referenced tools. Some of these tools are directly linked to OpenEBench (https://openebench.bsc.es/dashboard; part of the ELIXIR Tools Platform) where public reference datasets have been made available to the community for the benchmarking of genomic variant calling tools and pipelines. The Community also benchmarked and established a genomic variant calling pipeline that was integrated in the RD‐Connect Genome‐Phenome Analysis Platform (GPAP; https://platform.rd‐connect.eu) and, in collaboration with the Tools and Interoperability Platform, was adapted to GA4GH [https://www.ga4gh.org/] and Common Workflow Language (CWL) [https://www.commonwl.org/] standards to run on the WES‐TES GA4GH cloud [https://www.ga4gh.org/news/ga4gh‐wes‐api‐enables‐portable‐genomic‐analysis/].

The ELIXIR‐EXCELERATE Rare Disease Community also contributed to the definition of the FAIR Guiding Principles (Wilkinson *et al*, 2016) and generic services such as the DCAT‐based FAIR data point specification [https://github.com/FAIRDataTeam/FAIRDataPoint‐Spec], Bioschemas [https://bioschemas.org/] extensions, ontology services and their tailoring for RD research, in collaboration with the Interoperability Platform. This data FAIRification process has subsequently been applied to several RD registries (e.g. Osteogenesis imperfecta in collaboration with the Rizzoli Institute in Bologna, Italy, and vascular anomalies in collaboration with the Radboud Medical Centre and their registry software provider Castor EDC, the Netherlands), and is being further developed and scaled up through the European Joint Program Rare Disease with the aim to develop sustainable FAIRification services and integration in routine RD workflows for establishing a FAIR‐based virtual platform for rare disease multidisciplinary research.

## Processing and deposition data resources for biodiversity data from the marine metagenome

Metagenomic data are a relatively new source of genomic data derived from samples from a wide range of environments, ranging from marine and soil, to the human gut. Standards to process and deposit data, for assembly of metagenomic‐assembled genomes (MAGs), and their deposition into appropriate databases, have been lacking. As a first step to addressing this, and to increase the amount of data available from the marine environment, the ELIXIR Marine Metagenomics Community (with major involvement from EMBL‐EBI and ELIXIR—Norway, ELIXIR—France and ELIXIR—Italy) refined databases and tools specific to marine metagenomics and worked to provide better integration and compatibility across those workflows and tools. In collaboration with the Interoperability and Compute Platforms, the Community drove the development and use of CWL for the description of metagenomic analysis pipelines to increase transparency and reproducibility (e.g. https://github.com/EBI‐Metagenomics/pipeline‐v5). Furthermore, the use of these more formal tool and pipeline descriptions allows them to be rapidly repurposed to establish a transcriptome annotation pipeline (https://github.com/EBI‐Metagenomics/workflow‐is‐cwl), with the outputs forming the backbone of MetDB [http://metdb.sb‐roscoff.fr/metdb/], a new micro‐eukaryotic marine transcriptome database, which is being adopted within EOSC‐Life. The Community published best practices (ten Hoopen *et al*, 2017) that serve as a foundation for a community standard to enable reproducibility and better sharing of metagenomic datasets. In future, the Marine Metagenomics Community is planning to broaden its scope to focus on the microbiome as a whole, enabling a larger community to benefit from the workflows and tools developed through ELIXIR‐EXCELERATE.

## Principles emerging from the work of ELIXIR‐EXCELERATE

ELIXIR provides small amounts of funding to support infrastructure elements relating, for example, to the needs of particular Communities. The ELIXIR‐EXCELERATE use cases became the first members of the ELIXIR Communities. A number of projects funded by ELIXIR have worked to improve workflows to analyse data. As well as the work of the ELIXIR Marine Metagenomics Community, there is an ongoing effort to standardise workflows for fluxomics by the ELIXIR Metabolomics Community, a community that emerged within ELIXIR after the start of ELIXIR‐EXCELERATE. Underlying this activity is the adoption of new standards and technical developments for workflow description. A trailblazer for this was the adoption of CWL, used to describe workflows by the ELIXIR Marine Metagenomics Community. Additionally, work is taking place under the auspices of the ELIXIR Galaxy Community to improve Galaxy’s utility as a tool for reproducible analysis, including improving the use of software virtualisation using different container technologies.

Key to the reuse of data is ease of deposition into central databases, often Core Data Resources (CDRs) (Durinx *et al*, 2017) such as the ENA, and the adoption of metadata standards (including ontologies) to describe data and make it more understandable and reusable. Many ELIXIR Communities have undertaken work to improve deposition of data into central databases, notably the Plant Science, Marine Metagenomics, Metabolomics and Proteomics Communities. These efforts improve submission to a range of databases; not only ENA, but also MetaboLights [https://www.ebi.ac.uk/metabolights/] and PRIDE [https://www.ebi.ac.uk/pride/], and by doing so, make the data accessible to the broader scientific community. Major databases have their own standards for data description but many have evolved in response to new requirements from ELIXIR Communities.

In some cases, data cannot be readily consolidated in a central database. The reasons for this differ. For human data, it may not be possible or desirable for data to cross‐national borders for regulatory reasons. ELIXIR has addressed this by building on two interlocking solutions—the Federated EGA, linked to the central EGA archive, to allow local storage of data in a standard format combined with regulated sharing of metadata; and Beacons, which allow non‐identifiable identification of potentially useful datasets. In the case of distributed datasets such as those handled by the Plant Science Community, the barriers are more technical in nature, reflecting the huge disparity of the data to be described. In this case, there has also been a need to gain adoption of metadata standards to describe the provenance of datasets. The adoption of BrAPI was predicated on the adoption of the MIAPPE metadata standard by databases that wished to be part of the FAIDARE network. This multi‐layered approach provides an excellent example of how the development of a suite of standards can deliver reusable data to a community of researchers.

## How does ELIXIR’S work help the working scientist?

Much of ELIXIR’s work during the ELIXIR‐EXCELERATE project was directed to developing guidelines and approaches for the FAIRification of data in different aspects. It increased the findability of human, plant and marine metagenome data using a variety of infrastructures, either to deposit data in central databases, or to federate datasets when consolidation has not been a practical solution. Accessibility does not exclusively pertain to data, but also to other types of objects such as software tools, workflows and training materials. Therefore, ELIXIR has extended FAIRness of software resources via the bio.tools registry [https://bio.tools/], part of the ELIXIR Tools Platform, which makes descriptions of, and access to, research software resources easier and more standardised and provided the TeSS registry for training materials, training workflows and training events [https://tess.elixir‐europe.org/] which enables scientists to find and access training resources easily. FAIRsharing [https://fairsharing.org/] provides curated resources on data and metadata standards, enabling interoperability of datasets and software, both via registries and specifications that can be applied *at source* (e.g. Bioschemas and the DCAT‐based FAIR data point specification). This is the kind of work that is often invisible to many researchers in the life science arena, but it results in working data processing resources, and better described datasets that are more suitable for data reuse.

## Conclusions and future directions

The evolution of ELIXIR during the ELIXIR‐EXCELERATE project resulted in a mature infrastructure that benefits the European life science community at a number of levels and was an essential learning phase for ELIXIR. At the highest level, it drove the evolution of national bioinformatics communities by the formation of the national ELIXIR Nodes. Various themes have emerged across the broad range of activities improving the FAIRness of data and the software resources used to process and analyse that data. A lot was learnt about how to ensure data findability and accessibility using a variety of mechanisms, and about the hard work needed to make data and software interoperable. To build on this, ELIXIR has initiated a tools ecosystem that will integrate diverse research software descriptions through its registries such as bio.tools and Biocontainers [https://biocontainers.pro/], benchmarking through OpenEBench and integration of the workflows through the WorkflowHub registry [https://workflowhub.eu/]. To improve data discoverability, a key future development will be to widen the uptake of the Bioschemas standard which allows the discovery of datasets on the web, and via tailored tools.

Thanks to the developments during the ELIXIR‐EXCELERATE project, ELIXIR was able to quickly respond to the 2019–2020 SARS‐CoV‐2 pandemic (Blomberg & Lauer, 2020). For example, the ELIXIR Galaxy Community, with close links to the Tools, Training and Compute Platforms, has played a key role in the European efforts to identify potential therapeutic small molecules against the SARS‐CoV‐2 Spike protein [https://covid19.galaxyproject.org/cheminformatics/#background]. In collaboration with the Instruct‐ERIC ESFRI and the UK Diamond Light Source, the Galaxy Community provided distributed compute infrastructure for the implementation of rapid, parallel workflows to prioritise potential small molecules. This made use of the recently implemented PULSAR network, which enables a job execution system distributed across several European centres, allowing the scaling of the computing power of Galaxy instances over different resources. The Galaxy Community has also driven the development of a European network of accessible Galaxy servers [https://galaxyproject.eu/]. More broadly, ELIXIR has supported research into SARS‐CoV‐2 across its many Platforms [https://elixir‐europe.org/services/covid‐19], including facilitating the development of the COVID‐19 Disease Map [https://covid19map.elixir‐luxembourg.org/minerva/].

A key objective of ELIXIR is the long‐term sustainability of datasets and software. To stabilise datasets over the long term, a major aspect is to ensure stable funding of key databases and remove them from the usual funding cycle based on expectation of scientific innovation. Building on its development of its CDRs, a process developed during the ELIXIR‐EXCELERATE project, ELIXIR contributed to the establishment of the Global BioData Coalition [https://globalbiodata.org/], whose aim is to coordinate national funders worldwide to support major data resources. For software, ELIXIR sees the European Open Science Cloud as a key infrastructure for maintaining widely usable workflows, making them accessible for any life science scientist to use and, in the context of infrastructures such as Galaxy, to modify workflows to support individual needs. ELIXIR coordinates the EOSC‐Life project [https://www.eosc‐life.eu/], which aims to facilitate access to life science data, tools and workflows in the context of a hybrid cloud infrastructure, across a range of data types provided by the various life science ESFRI infrastructures.

ELIXIR’s work on workflows leading to data deposition has surfaced the importance of pre‐submission data management. To address this, ELIXIR coordinates the ELIXIR‐CONVERGE project [https://elixir‐europe.org/about‐us/how‐funded/eu‐projects/converge], bringing together infrastructure and national expertise in data management across its members. Capacity building is a key output of ELIXIR‐EXCELERATE, and its training activities, including its TeSS registry and standards for training courses, including post‐training follow‐up, continue to develop.

Human data remain a key priority for ELIXIR, which is achieved via its own technical developments, community coordination via its Human Data Communities and coordinating European engagement with initiatives such as GA4GH and B1MG (the Beyond One Million Genomes project) [https://b1mg‐project.eu/]. More broadly, the ELIXIR Community structure, which brings together experts in particular technical and scientific areas with the potential to carry out small projects to develop infrastructural components, is a key way for ELIXIR to learn what needs to be done in the future, and expand the areas in which the ELIXIR infrastructure is usable by different stakeholders.

In conclusion, since 2014, ELIXIR has evolved into a dynamic yet well‐developed infrastructure enabling state‐of‐the‐art life science research. ELIXIR combines technical and coordination activities both across Europe and globally. Its vision for the future is shaped by its constituent communities, both formal and informal, and is focussed on building a technical infrastructure to provide FAIR data and software, structures that deliver capacity building within its Nodes, and sustainability of the data and tools ecosystem upon which life science scientists increasingly rely.

## Author contributions

We confirm that the funders had no role in study design, data collection and analysis, decision to publish or preparation of the manuscript.

## Conflict of interest

The authors declare that they have no conflict of interest.

## Appendix 1

### ELIXIR‐EXCELERATE Community

Niklas Blomberg [N.B.] (0000‐0003‐4155‐5910), ELIXIR Hub, Wellcome Genome Campus, Hinxton, Cambridge, CB10 1SD, UK.

Søren Brunak [S.B.] (0000‐0003‐0316‐5866), Technical University of Denmark & University of Copenhagen.

Salvador Capella‐Gutierrez [S.C.‐G.] (0000‐0002‐0309‐604X), Barcelona Supercomputing Center (BSC).

Christine Durinx [C.D.] (0000‐0003‐4237‐8899), SIB Swiss Institute of Bioinformatics.

Chris T. Evelo [C.T.E.] (0000‐0002‐5301‐3142) Department of Bioinformatics ‐ BiGCaT, Research School NUTRIM, Maastricht University.

Carole Goble [C.G.] (0000‐0003‐1219‐2137; 0000‐0001‐7219‐632X), The University of Manchester.

Ivo Gut [I.G.] (0000‐0001‐7219‐632X) Centro Nacional de Análisis Genómico (CNAG‐CRG), Centre for Genomic Regulation, Barcelona Institute of Technology; Universitat Pompeu Fabra.

Jon Ison [J.I] (0000‐0001‐6666‐1520) Technical University of Denmark.

Thomas Keane [T.K.] (0000‐0001‐7532‐6898), European Bioinformatics Institute.

Brane Leskošek [B.L.] (0000‐0001‐5202‐2349) University of Ljubljana, Faculty of Medicine, ELIXIR‐SI.

Luděk Matyska [L.M.] (0000‐0001‐6399‐5453) Masaryk University; CESNET.

Johanna McEntyre [J.M.] (0000‐0002‐1611‐6935) European Bioinformatics Institute.

Célia Miguel [C.M.] (0000‐0002‐1427‐952X) Biosystems & Integrative Sciences Institute (BioISI), Faculdade de Ciências, Universidade de Lisboa, Portugal; iBET Instituto de Biologia Experimental e Tecnológica, Oeiras, Portugal.

Arcadi Navarro [A.N.] (0000‐0003‐2162‐8246) Institute of Evolutionary Biology (UPF‐CSIC), Department of Experimental and Health Sciences, Pompeu Fabra University, Barcelona Biomedical Research Park, Carrer del Dr. Aiguader 88, 08003 Barcelona, Spain; Catalan Institution of Research and Advanced Studies (ICREA), Passeig de Lluís Companys 23, 08010 Barcelona, Spain; CRG, Centre for Genomic Regulation, Barcelona Institute of Science and Technology (BIST), Carrer del Dr. Aiguader 88, 08003 Barcelona, Spain; Barcelonaβeta Brain Research Center (BBRC), Pasqual Maragall Foundation, Wellington 30, 08005, Barcelona, Spain.

Steven Newhouse [S.N.] (0000‐0003‐1531‐5198) European Bioinformatics Institute.

Tommi Nyrönen [T.N.] (0000‐0002‐5569‐5183) CSC ‐ IT Center for Science Ltd.

Patricia Palagi [P.P.] (0000‐0001‐9062‐6303) SIB Swiss Institute of Bioinformatics.

Bengt Persson [B.P.] (0000‐0003‐3165‐5344) NBIS (National Bioinformatics Infrastructure Sweden) and ELIXIR‐SE, Department of Cell and Molecular Biology, SciLifeLab, Uppsala University, Sweden.

Cyril Pommier [C.P.] (0000‐0002‐9040‐8733) Université Paris‐Saclay, INRAE, URGI, 78026, Versailles, France; Université Paris‐Saclay, INRAE, BioinfOmics, Plant bioinformatics facility, 78026, Versailles, France.

Jordi Rambla [J.R.] (0000‐0001‐9091‐257X) CRG, Centre for Genomic Regulation, Barcelona, Spain.

Marco Roos [M.R.] (0000‐0002‐8691‐772X) Leiden University Medical Center, Leiden, Netherlands.

Gabriella Rustici [G.R.] (0000‐0003‐3085‐1271), University of Cambridge, Cambridge, UK.

Andrew Smith [A.S.] (0000‐0003‐3085‐1271) ELIXIR Hub, Wellcome Genome Campus, Cambridge, UK.

Alfonso Valencia [A.V.] (0000‐0002‐8937‐6789) Barcelona Supercomputing Center (BSC), Barcelona, Spain; Institució Catalana de Recerca i Estudis Avançats (ICREA).

Celia van Gelder [C.G.](0000‐0002‐0223‐2329) DTL Dutch Techcentre for Life Sciences.

Jiri Vondrasek [J.V.] (0000‐0002‐6066‐973X) IOCB AS CR.

Nils Peder Willassen [N.P.W.] (0000‐0002‐4397‐8020) UiT The Arctic University of Norway.

Juan Arenas [J.A.] (0000‐0001‐5497‐8045), ELIXIR Hub, Wellcome Genome CampusHinxton, Cambridge, CB10 1SD, UK.

Helen Parkinson [H.P.] (0000‐0003‐3035‐4195) European Bioinformatics Institute.

Robert D. Finn [R.D.F.] (0000‐0001‐8626‐2148) European Bioinformatics Institute.

Sergi Beltran [S.B.] (0000‐0002‐2810‐3445) Centro Nacional de Análisis Genómico (CNAG‐CRG), Centre for Genomic Regulation, Barcelona Institute of Technology; Universitat Pompeu Fabra; Dep. Genetics, Microbiology and Statistics, Facultat de Biologia, Universitat de Barcelona.

Leslie Matalonga [L.M.] (0000‐0003‐0807‐2570) Centro Nacional de Análisis Genómico (CNAG‐CRG), Centre for Genomic Regulation, Barcelona Institute of Technology.

Hannah Hurst [H.H.] (0000‐0002‐1119‐9321), ELIXIR Hub, South Building, Wellcome Genome Campus, Hinxton, Cambridge, CB10 1SD, UK.

Paul Kersey [P.K.] (0000‐0002‐7054‐800X) European Bioinformatics Institute.

Ilkka Lappalainen [I.L.] (0000‐0001‐5762‐893X) CSC ‐ IT Center for Science Ltd.

Pascal Kahlem [P.K.] (0000‐0002‐8810‐444X), Scientific Network Management, Barcelona, Spain.

Gary Saunders [G.S.] (0000‐0002‐7468‐0008), ELIXIR Hub, South Building, Wellcome Genome Campus, Hinxton, Cambridge, CB10 1SD, UK.

Sirarat Sarntivijai [S.S.] (0000‐0002‐2548‐641X) ELIXIR Hub, South Building, Wellcome Genome Campus, Hinxton, Cambridge, CB10 1SD, UK.

Rachel Drysdale [R.D.] (0000‐0003‐3037‐0216), ELIXIR Hub, South Building, Wellcome Genome Campus, Hinxton, Cambridge, CB10 1SD, UK.

Jonathan Tedds [J.T.] (0000‐0003‐2829‐4584) ELIXIR Hub, South Building, Wellcome Genome Campus, Hinxton, Cambridge, CB10 1SD, UK. ^§^ Correction added on 15 March 2021, after first online publication: the author's name was changed from Johnathan Tedds to Jonathan Tedds.

Jeremy Lanfear [J.L.] (0000‐0002‐8007‐5568) ELIXIR Hub, South Building, Wellcome Genome Campus, Hinxton, Cambridge, CB10 1SD, UK.

Jennifer Harrow [J.H.] (0000‐0003‐0338‐3070) ELIXIR Hub, South Building, Wellcome Genome Campus, Hinxton, Cambridge, CB10 1SD, UK.
